# Bisphenol-A Abrogates Proliferation and Differentiation of C2C12 Mouse Myoblasts via Downregulation of Phospho-P65 NF-*κ*B Signaling Pathway

**DOI:** 10.1155/2024/3840950

**Published:** 2024-02-28

**Authors:** Chittipong Tipbunjong, Thanvarin Thitiphatphuvanon, Chumpol Pholpramool, Piyaporn Surinlert

**Affiliations:** ^1^Department of Anatomy, Division of Health and Applied Sciences, Faculty of Science, Prince of Songkla University, Songkhla 90110, Thailand; ^2^Faculty of Medicine, Kasetsart University, Bangkok 10900, Thailand; ^3^Department of Physiology, Faculty of Science, Mahidol University, Bangkok 10400, Thailand; ^4^Chulabhorn International College of Medicine, Thammasat University, Bangkok, Pathum-Thani 12120, Thailand; ^5^Thammasat University Research Unit in Synthesis and Applications of Graphene, Thammasat University, Pathum-Thani 12120, Thailand

## Abstract

Previous studies showed that bisphenol-A (BPA), a monomer of polycarbonate plastic, is leached out and contaminated in foods and beverages. This study aimed to investigate the effects of BPA on the myogenesis of adult muscle stem cells. C2C12 myoblasts were treated with BPA in both proliferation and differentiation conditions. Cytotoxicity, cell proliferation and differentiation, antioxidant activity, apoptosis, myogenic regulatory factors (MRFs) gene expression, and mechanism of BPA on myogenesis were examined. C2C12 myoblasts exposed to 25–50 *µ*M BPA showed abnormal morphology, expressing numerous and long cytoplasmic extensions. Cell proliferation was inhibited and was accumulated in subG1 and S phases of the cell cycle, subsequently leading to apoptosis confirmed by nuclear condensation and the expression of apoptosis markers, cleaved caspase-9 and caspase-3. In addition, the activity of antioxidant enzymes, catalase, superoxide dismutase, and glutathione peroxidase was significantly decreased. Meanwhile, BPA suppressed myoblast differentiation by decreasing the number and size of multinucleated myotubes via the modulation of MRF gene expression. Moreover, BPA significantly inhibited the phosphorylation of P65 NF-*κ*B in both proliferation and differentiation conditions. Altogether, the results revealed the adverse effects of BPA on myogenesis leading to abnormal growth and development via the inhibition of phospho-P65 NF-*κ*B.

## 1. Introduction

Skeletal muscle is the most abundant tissue of living organisms and plays a major role in body movement and metabolism. The skeletal muscle tissues are derived from mesoderm during embryonic development. Mesodermal cells become myoblasts, which are muscle precursor cells, under the regulation of myogenic regulatory factors (MRFs) including MyoD, myogenin, myogenic factor 5 (myf-5), and myogenic regulatory factor 4 (MRF4) that play significant roles in myogenesis through two crucial steps. First, myoblasts proliferate to increase cell numbers for muscle mass under the regulation of MyoD and myf-5. The subsequent step is myoblast differentiation, in which myoblasts differentiate into myocytes by expressing myosin heavy chain (MHC), the major structural protein in muscle. These myocytes eventually fuse together and mature into multinucleated myofibers under the control of myogenin and MRF4 [1].

At the later phase of myogenic development, some mesodermal cells give rise to satellite cells, which are important for muscle growth and regeneration after birth. The satellite cells are localized between muscle fibers in adult muscle. Upon muscle injury, the satellite cells are activated and expressed MRFs leading to muscle regeneration that involves the proliferation and differentiation of satellite cells and their survival and integration into functional myofibers [2, 3].

Defects in myogenesis and/or muscle regeneration lead to malformation or atrophy of skeletal muscle, which would affect locomotion. The rate of adult myogenesis is modulated by an array of exogenous and endogenous factors. Positive factors include exercise [4], growth factors [5, 6], and hormones [7, 8]. Conversely, negative factors that decrease myogenesis include aging [9], diseases [10, 11], and environmental toxicants [12, 13].

Bisphenol-A (BPA, 2,2′-bis(4-hydroxyphenyl) propane) is a monomer employed in the manufacturing of polycarbonate and epoxy resin. In turn, these materials are used worldwide for the production of many consumer products including plastic, internal coating of packaging, and medical devices. In particular, like other chemical monomers, numerous studies have reported the leaching of BPA from these materials [14]. Extreme temperature and pH conditions increase the rate of leaching [15]. BPA has been found to contaminate various products such as soft drinks [16], kinds of seafood [17], and even breast milk [18]. Moreover, the bioaccumulation of BPA in liver, muscle, and brain tissues has been reported [19]. Exposure to BPA gradually increases scientist's concern because it has been found in the urine of more than 90% of individuals in the United States, Germany, and Canada [20]. Another reason for concern is that it can easily pass the placental barrier and thus affect the developing embryo [21] and can cause human infertility in both male and female adults [22].

Toxicological and epidemiological studies show solid evidence for the effect of BPA on myogenesis, but its underlying mechanisms are poorly understood. Herein, we explored the effects of BPA on two crucial steps during myogenesis, i.e., myoblast proliferation and differentiation. We observed that BPA significantly inhibited myoblast proliferation leading to cellular apoptosis and markedly abrogated myoblast differentiation by inhibiting the expression of myogenic regulatory factor genes via downregulating the phosphorylation of JNK, p53, and P65 NF-*κ*B proteins.

## 2. Materials and Methods

### 2.1. Chemicals and Reagents

Otherwise indicated, chemicals and reagents for cell culture were purchased from Gibco (Life Technologies, Carlsbad, CA, USA). Bisphenol-A (BPA) and other basic chemicals were obtained from Sigma-Aldrich (Saint Louis, MO, USA). Catalase, superoxide dismutase, and glutathione peroxidase assay kits were from Elabscience (Houston, Texas, USA). Viva cDNA synthesis kit and Luna universal RT-qPCR were from Vivantis (Selangor Darul Ehsan, Malaysia) and New England Biolabs (Ipswich, MA, USA), respectively. The primary antibodies including anti-MHC (Millipore, Billerica, MA, USA), anti-caspase-9, anti-caspase-8, anti-caspase-3 (Cell Signaling Technology, Danvers, MA, USA), anti-myogenin, anti-phospho-p53, anti-JNK, anti-phospho-JNK, and anti-phospho-P65 NF-*κ*B (Santa Cruz Biotechnology, CA, USA) were also used in this study.

### 2.2. C2C12 Myoblast Cell Culture and Treatment

C2C12 myoblast cell line was purchased from American Type Culture Collection (ATCC; Manassas, VA, USA). Cells were grown in growth medium (GM) composed of Dulbecco's Modified Eagle's Medium (DMEM) supplemented with 10% fetal bovine serum (FBS) and 1% antibiotics at 37°C in a humidified 5% CO_2_ incubator. To test the effect of BPA on cell cytotoxicity and cell proliferation, the myoblasts were cultured in serum-free DMEM or GM containing BPA at 0–50 *µ*M for 24, 48, and 72 h. To test the effect on myogenic differentiation, approximately 80% of confluent cells were cultured in differentiation medium (DM; DMEM supplemented with 2% horse serum) containing BPA at 0–50 *µ*M for 3 days.

### 2.3. 3-(4,5-Dimethylthiazol-2-yl)-2,5-Diphenyltetrazolium Bromide (MTT) Assay

Cell cytotoxicity and cell proliferation were measured by MTT assay. Briefly, the treated cells were shifted to GM containing 0.5 mg/ml MTT and were then incubated at 37°C for 4 h. Then, the MTT solution was discarded and replaced with 100 *μ*l dimethyl sulfoxide. After constant agitation, the absorbance was determined using a microplate reader (Synergy HT; BioTek, USA) at 570–630 nm.

### 2.4. Cell Cycle Analysis

After 72 h of treatment, C2C12 cells were trypsinized and were then fixed with 70% ice-cold ethanol overnight at −20°C. After several washes with phosphate-buffered saline (PBS), the treated cells were incubated with ribonuclease A at 37°C for 30 min. The mixture was chilled on ice, and propidium iodide was directly added. The mixture was kept on ice in the dark for 15 min. The cell cycle stages of treated cells were determined and analyzed using BD FACSCanto™ flow cytometer (BD Biosciences) and BD FACSDiva version 6.1.1 software, respectively.

### 2.5. Protein Concentration Measurement

After treatment, myoblast cells were collected by trypsinization and washed with PBS. The protein was extracted using radio-immunoprecipitation assay (RIPA) buffer with a protease inhibitor cocktail. The lysate was centrifuged at 14,000 rpm, 4°C for 20 min, and protein concentration was determined using a bicinchoninic acid (BCA) protein assay kit.

### 2.6. Antioxidant Enzymes Assays

After treatment, treated cells were harvested by trypsinization and were lysed in PBS by repeated freeze-thaw method. The enzyme activities of superoxide dismutase (SOD), catalase (CAT), and glutathione peroxidase (GPx) were measured using commercial kits following the manufacturer's instructions.

### 2.7. Western Blot

Equal amounts of proteins were subjected to sodium dodecyl-sulfate polyacrylamide gel electrophoresis (SDS-PAGE) to separate proteins by size. The proteins were transferred onto a polyvinylidene difluoride (PVDF) membrane using the semidry machine. The membrane was probed with the desired antibodies for 1 h at room temperature (RT). After several washes, the membrane was incubated with appropriate horseradish peroxidase-conjugated secondary antibody for another 1 h at RT. The protein expression was visualized with ECL Western blotting detection reagent under the gel documentation system (BioSpectrum AC Chemi HR 410). The protein band intensity was measured by ImageJ software.

### 2.8. Immunofluorescence Staining

After treatment, treated cells were washed with PBS and fixed with ice-cold methanol for 10 min. After several washings, the fixed cells were allowed to rehydrate in PBS for at least 30 min. Cells were permeabilized and blocked with 5% normal goat serum and 0.3% Triton X-100 in PBS at RT for 1 h. Then, the cells were incubated with mouse monoclonal anti-MHC for 1 h. After several washes with PBS, cells were incubated with appropriate secondary antibody conjugated with fluorescein isothiocyanate (FITC) and Hoechst 33342 at RT for 45 min. The fluorescence signal was observed under a fluorescence microscope (Olympus IX73).

### 2.9. Quantitative Real-Time Polymerase Chain Reaction (qPCR)

After treatments, treated cells were harvested by trypsinization and subjected to RNA extraction. The RNA was converted to cDNA using the cDNA synthesis kit. The changes in gene expression were analyzed by real-time PCR using BioRad CFX96 Touch Real-Time PCR machine. The data were calculated using the 2^−ΔΔCt^ method. Primers used in this study were as follows: MyoD; 5′-ATGATGACCCGTGTTTCGACT-3′ and 5′-CACCGCAGTAGGGAAGTGT-3′,Myf-5; 5′-GCCTTCGGAGCACACAAAG-3′ and 5′-TGACCTTCTTCAGGCGTCTAC-3′, myogenin; 5′-GAGACATCCCCCTATTTCTACCA-3′ and 5′-GCTCAGTCCGCTCATAGCC-3′, MRF4; 5′- CTGAAGCGTCGGACTGTGG-3′ and 5′-ATCCGCACCCTCAAGAATTTC-3′, GAPDH; 5′-TGCGACTTCAACAGCAACTC-3′ and 5′-GCCTCTCTTGCTCAGTGTCC-3′ [23, 24].

### 2.10. Statistical Analysis

All experiments were performed independently, and results were given as mean ± SEM. The data were compared using one-way analysis of variance (ANOVA) followed by Tukey's multiple comparison test with GraphPad Prism version 5.00. Statistical differences were displayed as follows: ns—not significant; ^*∗*^*P* < 0.05; ^*∗∗*^*P* < 0.01; ^*∗∗∗*^*P* < 0.001.

## 3. Results

### 3.1. BPA Inhibited C2C12 Myoblast Proliferation

**Figure 1:**
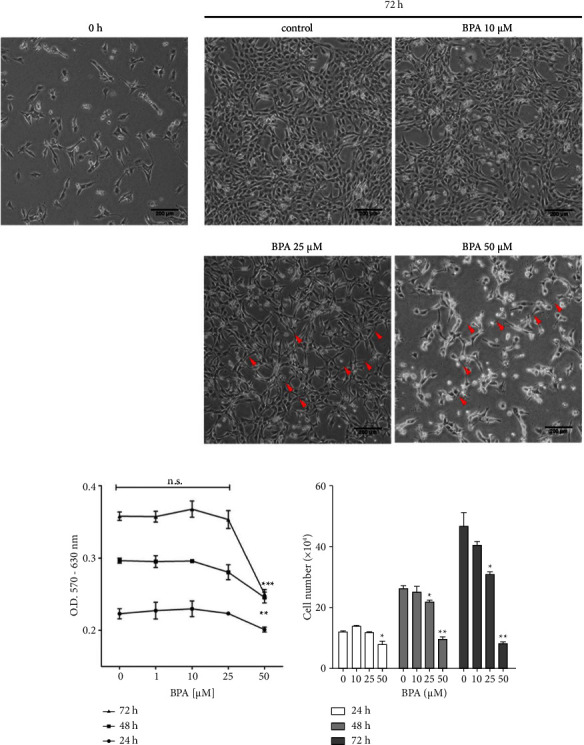
BPA inhibited C2C12 myoblast cell proliferation. C2C12 myoblast cells were cultured in a growth medium in the absence or presence of BPA at the indicated concentrations for 24, 48, and 72 h. Photomicrographs of cell morphology before and after BPA treatments (a). Cell viability was assessed by MTT assay (b) and cell counting (c). Arrowheads indicate cytoplasmic extension; n s.: not significant; ^*∗*^*P* < 0.05; ^*∗∗*^*P* < 0.01; ^*∗∗∗*^*P* < 0.001 compared to the control group. Scale bar = 200 *µ*m.

In this study, the cytotoxic effect of BPA on C2C12 myoblasts was assessed by observing cell morphology and MTT assay. After exposure to BPA at the indicated concentrations for 72 h, the morphology of the cells in control and in 10 *µ*M BPA-treated groups appeared flat, star-shaped, or fusiform, which represents the normal morphology of C2C12. However, increases in BPA concentration led to abnormal morphology: 25 *µ*M BPA treatment caused numerous and long cytoplasmic extensions whereas myoblasts in 50 *µ*M BPA treatment became round with fewer cytoplasmic extensions and started to detach from the culture dish surface (Figure 1(a)). Following 24, 48, and 72 h exposures to BPA at the indicated concentrations, discernible toxicity was observed at 50 *µ*M from 48 h exposure compared to the untreated control (Figure 1(b)). An equal absorbance value of 50 *µ*M BPA at 48-h and 72-h treated group suggests an inhibition of cell proliferation. To confirm this, cell counting was performed. Indeed, cell numbers in the 50 *µ*M BPA-treated groups at 24, 48, and 72 h showed no significant differences. In addition, 50 *µ*M BPA decreased cell numbers by 24 h compared to control. Also, by 48 h exposure to 25 *µ*M and 50 *µ*M BPA, the number of myoblasts was significantly decreased (Figure 1(c)). These results clearly demonstrate that BPA significantly inhibited C2C12 myoblast cell proliferation in both dose- and time-dependent manners.

### 3.2. BPA Induced Cell Cycle Arrest at subG1 Phase and Reduced Antioxidant Enzyme Activity

**Figure 2:**
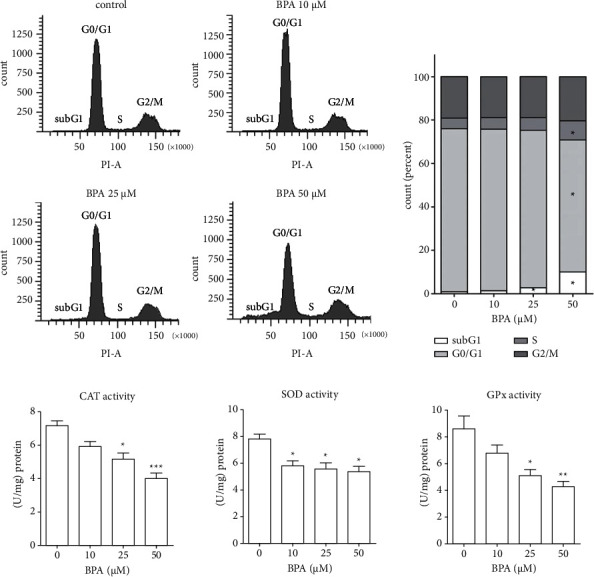
BPA induced cell cycle arrest and reduced antioxidant enzyme activity. C2C12 myoblast cells were cultured in a growth medium in the absence or presence of BPA for 72 h. The treated cells were collected, stained with propidium iodide, and subjected to flow cytometry for cell cycle analysis. Cell cycle distribution graph (a) and percentage of cell population in subG1, G0/G1, and S and G2/M phases (b). The treated cells were subjected to protein extraction and antioxidant enzyme activity determination (c). ^*∗*^*P* < 0.05, ^*∗∗*^*P* < 0.01; ^*∗∗∗*^*P* < 0.001 compared to the control group.

To further ascertain the inhibition of proliferation by BPA, cell cycle distribution was assessed by flow cytometry. Treatments of C2C12 myoblasts with BPA (25 and 50 *µ*M) for 72 h significantly increased the percentage of cell population in the subG1 phase (2.80 ± 0.29 and 10.05 ± 0.32, respectively) compared to untreated control (0.83 ± 0.19). An accumulation of cells at the subG1 phase led to a significant decrease in the percentage of cell population in the G0/G1 phase (Figures 2(a) and 2(b)). Furthermore, the percentage of cells in the S-phase after 50 *µ*M BPA treatment was significantly higher than those in other groups reflecting an accumulation of cells at this phase. In addition, the activity of antioxidant enzymes including superoxide dismutase (SOD), catalase (CAT), and glutathione peroxidase (GPx) after BPA treatment was measured. The results showed significant decreases in all antioxidant enzyme activities being tested in a dose-dependent manner (Figure 2(c)). Treatments with BPA at 25 *µ*M and 50 *µ*M significantly decreased CAT, SOD, and GPx activities compared to the nontreatment group.

### 3.3. BPA Induced Cellular Apoptosis

**Figure 3:**
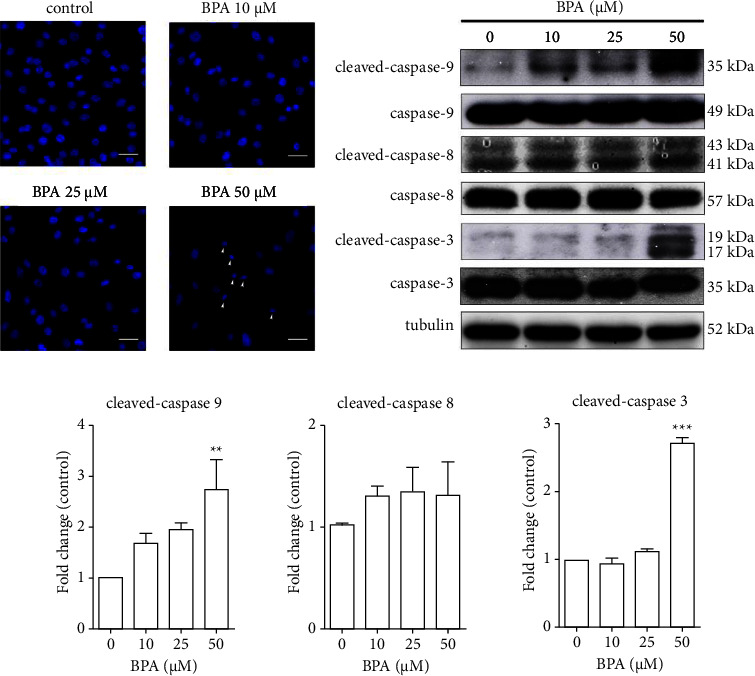
BPA induced cellular apoptosis. C2C12 myoblast cells were cultured in the absence or presence of BPA at the indicated concentrations for 72 h. Photomicrographs of treated cells stained with Hoechst (blue) to visualize nuclear morphology (a). Total proteins were subjected to Western blot analysis with anti-caspase-3, anti-caspase-8, and anti-caspase-9 antibodies (b) and expressed as relative band intensity to tubulin (c). ^*∗∗*^*P* < 0.01; ^*∗∗∗*^*P* < 0.001 compared to the control group. Scale bar = 20 *µ*m.

Cellular apoptosis is usually due to an imbalance between oxidant molecules and antioxidant enzymes, which protect the cells from radical-mediated damage. To investigate whether the reduction of antioxidant enzyme activity subsequently causes apoptosis, nuclear fragmentation and apoptotic markers were assayed. As expected, Hoechst staining exhibited round or oval nuclei with homogeneous chromatin in the untreated and 10–25 *µ*M BPA-treated groups, whereas cells exposed to 50 *µ*M BPA showed a reduction in nuclear size and chromatin condensation, although nuclear fragmentation could not be found (Figure 3(a), arrowheads). Western blot analysis also revealed significant expression of apoptotic markers, cleaved caspase-9 and cleaved caspase-3, but not cleaved caspase-8, at 50 *µ*M BPA treatment compared to untreated control (Figures 3(b) and 3(c)).

### 3.4. BPA Suppressed C2C12 Myoblast Differentiation

**Figure 4:**
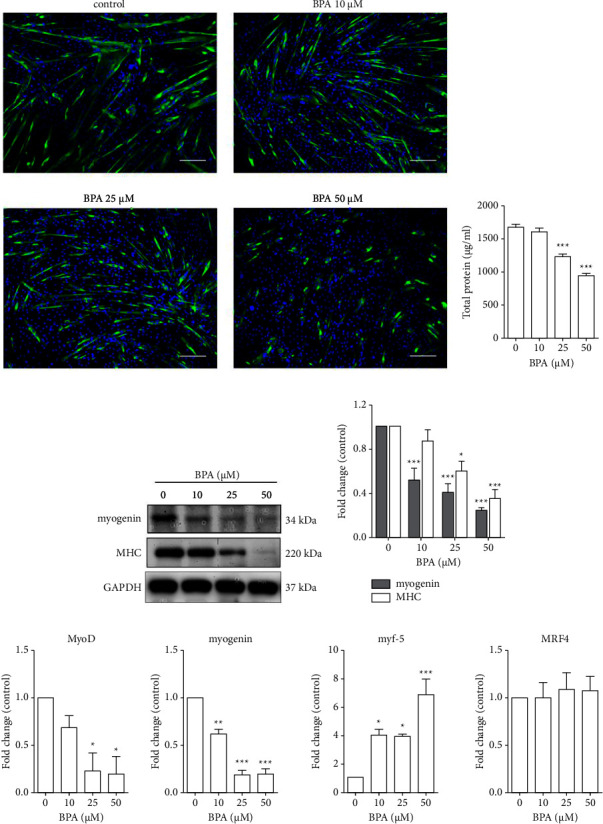
BPA suppressed C2C12 myoblast differentiation. Confluence C2C12 myoblast cells were cultured in differentiation conditions in the absence or presence of BPA for 72 h. Photomicrograph of immunofluorescence staining for MHC protein (green) and nuclei (blue) in differentiated myotubes (a). Total proteins were extracted and measured by BCA protein assay kit (b). Proteins were subjected to Western blot analysis with anti-MHC and anti-myogenin antibodies and expressed as relative band intensity to tubulin (c) and (d). Total RNA was extracted and subjected to real-time PCR with specific primers for the myogenic regulatory factors gene. Gene expression levels were expressed as fold change compared to the control group (e). ^*∗*^*P* < 0.05; ^*∗∗*^*P* < 0.01; ^*∗∗∗*^*P* < 0.001 compared to the control group. Scale bar = 200 *µ*m.

Effects of BPA exposure during C2C12 myoblast differentiation were assessed by measuring the total proteins, MHC, and myogenin protein expression as markers for myoblast differentiation. Following a 72 h exposure to BPA in differentiation condition, immunofluorescence staining for MHC was performed. Numerous large and elongated myotubes were present in the 10 *µ*M BPA treatment group, which was similar to the untreated control group. However, BPA treatment at 25 *µ*M caused smaller and shorter myotubes compared to the untreated control. Exposure to 50 *µ*M BPA nearly abolished myoblast differentiation, myotubes were rare and myocytes were scattered and stained positive for MHC but contained only 1-2 nuclei (Figure 4(a)). Consistent with the immunofluorescence staining, after treatment with 25 and 50 *µ*M BPA, total protein synthesis was significantly decreased by 25% and 43%, respectively (Figure 4(b)). In addition, Western blot analysis further confirmed significant decreases in both MHC and myogenin protein expression in 25 and 50 *µ*M treated groups compared to control, which explains the lower level of myoblast differentiation (Figures 4(c) and 4(d)). Since myogenesis is orchestrated by myogenic regulatory factor genes, we hypothesized that BPA may inhibit myoblast differentiation by suppressing the expression of these genes. The results showed that, after 72 h in differentiation condition with BPA at 25–50 *µ*M, the MyoD and myogenin expression levels were downregulated, being only about one-third of the control group. Conversely, the expression level of myf-5 was significantly increased in a dose-dependent manner, being nearly 4 folds at 10–25 *µ*M and 7 folds at 50 *µ*M BPA. Interestingly, MRF4 expression level did not change after treatment with BPA at all concentrations (Figure 4(e)).

### 3.5. BPA Suppressed Myogenesis via Downregulation of Phospho-P65 NF-*κ*B

**Figure 5:**
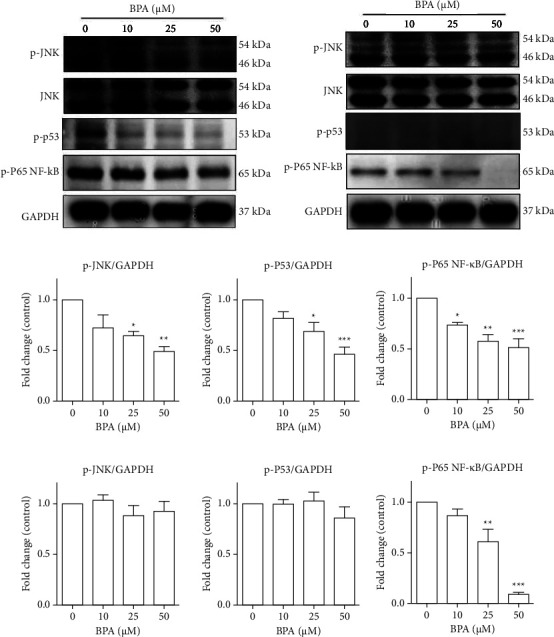
Molecular mechanism of BPA in C2C12 myoblast proliferation and differentiation. C2C12 myoblasts were cultured in either growth medium (GM) or differentiation medium (DM) in the absence or presence of BPA at the indicated concentration for 72 h. Total proteins were extracted and subjected to Western blot analysis with anti-phospho-JNK, anti-phospho-p53, and anti-phospho-P65 NF-*κ*B antibodies. The activation of each phosphorylated protein was expressed as fold change compared to the control group. ^*∗*^*P* < 0.05; ^*∗∗*^*P* < 0.01; ^*∗∗∗*^*P* < 0.001 compared to the control group. (a and c) Proliferation and (b and d) differentiation conditions.

Following treatment with 25–50 *µ*M BPA in proliferation condition for 72 h, the activation of p-JNK, p-p53, and p-P65 NF-*κ*B proteins significantly decreased compared to untreated control (Figures 5(a) and 5(c)). In contrast, in the differentiation condition for 72 h, a significant decrease in the activation of p-P65 NF-*κ*B protein only was found, while p-JNK and p-p53 were not suppressed compared to untreated control (Figures 5(b) and 5(d)).

## 4. Discussion

In the present study, we reported the adverse effect of BPA at the doses 1–50 *µ*M on C2 C12 mouse myoblast cell proliferation and differentiation in both a dose- and a time-dependent manner. The dosage that is cytotoxic (50 *µ*M) and that inhibits proliferation and differentiation (25–50 *µ*M) was much lower than those reported to induce toxicity in human bone mesenchymal stem cells (250 *µ*M) [25] and to inhibit cell proliferation in human fetal lung fibroblasts (100 *µ*M) [26]. Previous studies have also shown that BPA inhibited the proliferation of many cell types including neural stem cells [27] and colonic epithelial cells [28]. In contrast, the enhancement of cell growth by BPA has been reported in several cell lines: normal human mammary epithelial cells [29], human normal breast cells (HBL-100) [30], and breast cancer cells (MCF-7 and SkBr3) [31]. This discrepancy may be attributed to the variation in exposure time and cell type being used. It has been proposed that the action of BPA on cell proliferation is mediated through estrogen receptors (ERs) [30, 32], in concordance with the existence of ERs in myoblast cells [33, 34]. The ERs mediate the antiproliferative effect of BPA by interaction with epidermal growth factor receptor (EGFR) causing a reduction in cyclin D1 expression and upregulation of cell proliferation inhibitors p21 and p27 [32].

On the other hand, antioxidant enzymes (SOD, CAT, and GPx) act as a defense network against excess production and accumulation of intracellular reactive oxygen species (ROS) by neutralization process [35]. The imbalance between ROS level and antioxidant enzyme activity will lead to oxidative stress which, in turn, stimulates the expression of apoptotic molecules by an intrinsic pathway [36]. Treatment with BPA has been reported to induce intracellular ROS through both enzymatic and nonenzymatic formation of phenoxyl radicals [37] causing oxidative stress [38]. A lowering of antioxidant enzyme (SOD, CAT, and GPx) activities after BPA treatment in our experiment is likely due to the excess formation of free radicals under the oxidative stress. Exposure to BPA decreased antioxidant enzyme activities not only in myoblasts but also in plasma [39], liver [40], kidney, and testes [41] leading to different endpoints.

Of interest, our finding revealed changes in the morphology of the treated cells from flat, star-shaped to round with fewer cytoplasmic extensions. This is in concordance with the toxicological study which showed that exposure to toxic chemicals stimulated loss of cellular characteristic morphology, changes in cell shape, development of elongated cytoplasmic extensions/gripping spicules, and cell detachment [42]. The number of cytoplasmic extensions and cell detachment increased proportionally to toxic chemical concentrations [43, 44]. These phenomena could be explained by altering or damaging cell membrane structure leading to changes in the capacity of cells to adhere to the basement membrane.

In particular, BPA exposure was shown to stimulate cell cycle arrest at subG1 and S phases. These cells underwent cellular apoptosis by activating the expression of cleaved caspase-9 and cleaved caspase-3 proteins. Treatment with BPA has been reported to affect the cell cycle by mediating cell cycle arrest at the G1 phase in prostate cancer cells through activation of the EGFR/ERK/p53 signaling pathway [32] and in human lung fibroblasts by inducing double-strand breaks (DSB)-ataxia telangiectasia mutated (ATM)-p53 signaling [26]. The signaling pathway responsible for the suppression of cell cycle in myoblasts by BPA is not known and warrants future studies. Beyond the cell cycle arrest, it is evident that BPA exposure triggers the cleaving of procaspase-3 and procaspase-9 into cleaved caspase-3 and cleaved caspase-9, respectively. The cleaved caspase-3 is the primary activator of DNA fragmentation by inactivating DNA fragmentation factor 45 (DFF45)/inhibitor of caspase-activated DNase (ICAD) protein, in turn leading to cellular apoptosis [45]. The expression of cleaved caspase-9 confirmed that BPA induced apoptosis, in part, through the mitochondrial pathway [46]. Increases of ROS by BPA [37, 38] can cause mitochondrial membrane potential changes leading to the leak of cytochrome c into the cytoplasm. Cytochrome c can bind with Apaf-1 finally leading to caspase-9 activation [46].

Regarding myogenic differentiation, exposure to BPA abolished myoblast differentiation into multinucleated myotubes. Previous studies have also reported the effect of BPA on the differentiation of many cell types including neural stem cells [27], bone-marrow-derived mesenchymal stem cells [41], and germ cells [47]. This may be caused by the activation of ER after BPA treatment and then recruiting both ER genomic and nongenomic signaling pathways leading to aberrant cell biology [41]. It has also been reported that BPA reduced the synthesis of several proteins in human granulosa cells [48], which is in accordance with the present study that total protein concentration was decreased by 50%. A previous study suggested that this effect may be caused by a decrease in the expression of translation elongation factor proteins following BPA treatment [49]. Treatment with low concentrations of BPA not only causes changes in protein synthesis but also induces changes in protein expression profiles [49], which, in turn, affect various cell types at different endpoints.

Exposures of myoblasts to several substances have been reported to attenuate MRF gene expression subsequently leading to impaired myogenic differentiation. For example, lipopolysaccharide (LPS) significantly decreases MyoD and myogenin expression [50], and arsenic suppresses myogenin expression in both *in vitro* and *in vivo* models [51]. Similarly, our experiment showed that treatment with BPA significantly suppressed both MyoD and myogenin expressions leading to impaired myogenic differentiation. The function of MRF4 is still unclear, but several publications have revealed its role in terminal differentiation. Its expression is upregulated at the late stage of differentiation when myogenin reaches the maximum level and gradually decreases [52]. Myf-5 expression is activated in muscle precursor cells which have a distinct role in muscle cell lineage determination and proliferation [53]. This factor is not upregulated during myoblast differentiation [52]. The high level of myf-5 expression in our experiment may be due to the downregulation of MyoD, which, in turn, maintains the proliferative status of myoblasts expressing myf-5. Another explanation is that the low level of myoblast differentiation after BPA treatment leads to a low myotube to myoblast ratio. These two cell populations showed differential expression of myf-5: myf-5 expression is downregulated in multinucleated myotubes whereas the residual myoblasts called quiescent “reserve cells” still carry on expressing myf-5 [52].

Exposure to BPA provoked toxicity during myoblast proliferation and differentiation by downregulating the expression of p-P65 NF-*κ*B protein. As BPA has been reported to mimic estrogen [30, 32] together with the existence of ER in myoblast cells [33, 34], it is possible that BPA provoked toxicity through ER. The activation of ER has been reported to suppress NF-*κ*B activity [54]. Inhibition of the NF-*κ*B signaling pathway has been reported to induce apoptosis and suppress proliferation of human fibroblast-like synovial cells [55] since the NF-*κ*B mediated antiapoptotic function [56]. Accordingly, JNK and P53 proteins may contribute to cell cycle arrest and apoptosis under the influence of NF-*κ*B. Data from gain- and loss-of-function approaches revealed the cross-talk between NF-*κ*B and p53, and p53 was necessary for the NF-*κ*B-mediated gene expression [57]. Besides, JNK has been shown to play a crucial role in cell proliferation, and the balance of JNK signaling will determine whether the cells are committed to proliferation or programmed cell death [58]. However, under the extended differentiation condition, activation of NF-*κ*B activity has been reported as a positive regulator of myoblast differentiation by stimulating MHC and myogenin expressions [59, 60]. BPA exposure may impair NF-*κ*B activity via ER-Akt signaling since BPA treatment has been reported to suppress Akt signaling in myoblast leading to suppression of myoblast differentiation [13]. In addition, Akt has been shown to mediate the regulation of NF-*κ*B activity by inducing phosphorylation and subsequent degradation of inhibitor of *κ*B (I*κ*B) [61]. Moreover, inhibition of NF-*κ*B activity has also been reported to interfere with the expression of myogenic regulatory factors [60]. However, the relationship between NF-*κ*B, JNK, and P53 proteins in myoblast exposed to BPA in both proliferation and differentiation conditions needs further elucidation.

## 5. Conclusions

This study addresses the effects of BPA on myoblast proliferation and differentiation, providing a better understanding of the adverse effects of the environmental contaminant BPA. In this study, we demonstrated that exposure to BPA significantly inhibited myoblast proliferation by stimulating cell cycle arrest, which, in turn, led to cellular apoptosis. In addition, exposure to BPA during myogenic differentiation suppressed myoblast differentiation and muscle protein synthesis. The inhibitory effect on myoblast differentiation was associated with modification of myogenic regulatory factor gene expression, which could occur via inhibition of phosphorylation of P65 NF-*κ*B. It is concluded that BPA impairs the myogenesis of muscle progenitor/stem cells. However, this research was conducted in *in vitro* cell culture system which has limitations for long-term experiments, but a higher concentration of BPA than the normal range found in living organisms could be used to see the effects. Thus, the cell culture system cannot fully replicate the complexities of living organisms. Further studies in *in vivo* system are, therefore, required to confirm and explore the toxic effects of BPA on myoblast cell proliferation and differentiation.

## Data Availability

The datasets used to support the findings of this study are available from the corresponding author upon request.
